# Profiles of Willingness to Use Pre-Exposure Prophylaxis Modalities and an HIV Vaccine Among Sexual and Gender Minority Individuals in Brazil, Mexico, and Peru: Cross-Sectional Online Survey

**DOI:** 10.2196/75753

**Published:** 2026-02-09

**Authors:** Jazmin Qquellon, Kelika A Konda, Oliver Elorreaga, Hamid Vega-Ramirez, Centli Guillén-Díaz-Barriga, Dulce Díaz-Sosa, Brenda Hoagland, Juan V Guanira, Marcos Benedetti, Cristina Pimenta, Beatriz Grinsztejn, Carlos F Caceres, Valdilea G Veloso, Thiago S Torres

**Affiliations:** 1 Centro de Investigación Interdisciplinaria en Sexualidad, Sida y Sociedad Universidad Peruana Cayetano Heredia Lima Peru; 2 Division Disease Prevention, Policy and Global Health, Department of Population and Public Health Sciences Keck School of Medicine University of Southern California Los Angeles, California, CA United States; 3 Division of Epidemiology and Psychosocial Research Instituto Nacional de Psiquiatría Ramón de la Fuente Muñiz Mexico City Mexico; 4 Faculty of Psychology National Autonomous University of Mexico Mexico City Mexico; 5 Department of Health Sciences Metropolitan Autonomous University-Lerma (UAM-L) Lerma de Villada Mexico; 6 Instituto Nacional de Infectologia Evandro Chagas Fundação Oswaldo Cruz Rio de Janeiro Brazil

**Keywords:** HIV prevention, vaccine, men who have sex with men, transgender women, cabotegravir, lenacapavir

## Abstract

**Background:**

HIV incidence continues to disproportionately affect sexual and gender minority (SGM) individuals in Latin America. Pre-exposure prophylaxis (PrEP), including long-acting products, urgently need scaling up in the region. Understanding PrEP modality preferences can help design effective implementation.

**Objective:**

This study examined willingness to use different PrEP modalities and an HIV vaccine among SGM individuals aged 18 years or older from Brazil, Mexico, and Peru and factors associated with willingness to use 4 PrEP modalities.

**Methods:**

We conducted a cross-sectional online survey in 2021; participants were recruited via apps (Grindr and Hornet) and social media (Facebook, Instagram, and WhatsApp). We used multivariate Poisson regression with robust variance (α=.05) to estimate prevalence ratios, identifying differences in willingness to use daily oral, event-driven oral, monthly oral, and bimonthly injectable PrEP. Models were constructed for each PrEP modality and adjusted for age, country, race, education, income, HIV risk score, HIV testing, and HIV risk perception. Variables were retained in the final adjusted models regardless of statistical significance.

**Results:**

Among 16,951 respondents, 10,385 (61.3%) were Brazilian, 4996 (29.5%) Mexican, and 1570 (9.3%) Peruvian. Median age was 32 (IQR 26-39) years. Among the total respondents, 12,621 (74.4%) were willing to use monthly oral PrEP; 11,153 (65.8%) daily oral PrEP; 10,212 (60.2%) bimonthly injectable PrEP; and 14,044 (82.8%) an HIV vaccine. Only 6442 (38%) were willing to use event-driven oral PrEP. In Brazil, 6082 (66.8%) were willing to use daily oral, 3162 (36.1%) event-driven, 7640 (74.9%) monthly oral, and 6450 (63.3%) injectable PrEP; in Mexico, 3242 (67.8%) daily oral, 1958 (41%) event-driven, 3728 (76.5%) monthly oral, and 2805 (57.5%) injectable PrEP; in Peru, 799 (53.1%) daily oral, 584 (39.9%) event-driven, 830 (62.5%) monthly oral, and 610 (45.9%) injectable PrEP. In multivariable models, willingness to use each of the 4 PrEP modalities was positively associated with high self-perceived HIV risk (adjusted prevalence ratios [aPRs] 1.10-1.25) and higher HIV Incidence Risk Index scores (aPRs 1.08-1.22). Having lower education was associated with lower willingness for monthly oral and bimonthly injectable PrEP (aPR=0.93, 95% CI 0.89-0.97 and aPR=0.94, 95% CI 0.90-0.99, respectively). Never having been tested for HIV and testing more than 6 months ago were associated with lower willingness for daily oral PrEP (aPR=0.89, 95% CI 0.83-0.95 and aPR=0.95, 95% CI 0.91-0.99, respectively) and bimonthly injectable PrEP (aPR=0.80, 95% CI 0.74-0.86 and aPR=0.90, 95% CI 0.86-0.94, respectively).

**Conclusions:**

Our results suggest a strong preference for long-acting formulations, including monthly oral and bimonthly injectable, among SGM individuals in Latin America. Further research is needed to address gaps in the understanding of prevention modalities. As additional PrEP modalities are included in HIV prevention programs, the development of accessible tools and community-based strategies will be essential to support informed PrEP choices and ensure equitable implementation across the region.

## Introduction

In 2024, an estimated 2.5 million people were living with HIV in Latin America [1]. Latin America is one of the few regions where HIV incidence has risen, with a 13% increase from 2010 to 2024 [2]. The incidence of HIV remains disproportionately higher among sexual and gender minority (SGM) individuals, who accounted for 66% of cases in Latin America in 2022. HIV prevalence among gay, bisexual, and other men who have sex with men (MSM) and transgender persons aged 15 to 49 years (2019-2023) was 10% and 9.5%, respectively [3]. Additionally, a study conducted in Brazil and Peru found high annualized HIV incidence among SGM individuals not using pre-exposure prophylaxis (PrEP; 3.9%, 95% CI 2.9-4.9), with higher incidence among participants from Peru, those aged 18 to 30 years, and those reporting lower incomes [4].

Currently, the World Health Organization (WHO) recommends daily or on-demand oral emtricitabine 200 mg and tenofovir disoproxil fumarate 300 mg (FTC/TDF), long-acting injectable cabotegravir (CAB-LA), long-acting injectable lenacapavir (LEN), and the dapivirine vaginal ring as effective HIV PrEP modalities [5]. As of July 2024, a total of 152 countries worldwide had adopted WHO recommendations on PrEP into their national guidelines, and 12 countries had adopted policies for CAB-LA [6]. In Latin America, oral PrEP was implemented as part of public policy in most countries, with Brazil initiating the provision of PrEP through the public health system (Sistema Único de Saúde [SUS]) in 2017, Mexico in 2021, and Peru in 2023 [7-9]. In 2024, a total of 286,862 persons were using PrEP in the region, with Brazil accounting for 58% of them [10]. In addition to regional disparities, more is needed to reach those who are at higher vulnerability to HIV. During the ImPrEP study, the largest PrEP implementation study in Latin America, younger participants, those who were Black or of mixed race (*pardo* or *mestizo*), those with lower educational attainment, and transgender women had higher HIV incidence and increased odds of discontinuing PrEP, lower adherence, and reduced persistence [11-13]. Similarly, PrEP uptake and persistence are worse among younger, Indigenous, and Black Brazilians according to Brazilian national data [14]. In response to implementation concerns with existing oral PrEP regimens, long-acting products need to be offered and accessible to those who need them most.

In 2021, CAB-LA was shown to be a highly effective alternative to daily oral FTC/TDF for HIV prevention among cisgender women, cisgender MSM, and transgender women [15,16]. Recently, subcutaneous LEN, administered twice yearly, was superior to FTC/TDF among cisgender women and SGM individuals in the phase 3 PURPOSE 1 and PURPOSE 2 clinical trials [17,18]. The frequency of administration of CAB-LA and LEN, bimonthly or biannually, respectively, is attractive for people who have difficulty adhering to an oral pill regimen. However, barriers to implementing injectable PrEP must be considered, including cost-effectiveness, staffing and other implementation constraints, acceptability and preferences among potential users, factors influencing timely attendance at injection visits, the prolonged subtherapeutic pharmacokinetic effect, and the feasibility of alternative administration sites [19,20]. While CAB-LA and LEN are not widely available in Latin America, Brazil is conducting the ImPrEP CAB Brasil and the ImPrEP LEN Brasil studies to evaluate the implementation of CAB-LA and LEN for SGM individuals aged 18 to 30 years in the public health system [21-23].

Different PrEP technologies, such as monthly oral, infusion of monoclonal antibodies, topical microbicides, and other long-acting products, are being developed [20,24]. For instance, subdermal implants have several advantages, such as easy removal; biodegradability; minimal health care system interaction; lower daily dosage; and more consistent, predictable drug release [20,25]. Islatravir delivered through a subdermal implant demonstrated promise as a potentially effective and well-tolerated approach for HIV prevention [26].

PrEP urgently needs to be scaled-up among SGM individuals in Latin America, including the provision of long-acting products, such as CAB-LA and LEN [3]. In this sense, understanding the willingness to use PrEP modalities could provide information for future adaptations or implementation of HIV prevention policies. We aimed to describe willingness to use different PrEP modalities and an HIV vaccine among SGM individuals in Brazil, Mexico, and Peru. We also assessed factors associated with willingness to use 4 PrEP modalities (daily oral, event-driven oral, monthly oral, and bimonthly injectable).

## Methods

### Study Design

A cross-sectional online survey was conducted between April and August 2021 among individuals who self-identified as SGM individuals and aged 18 years or older from Brazil, Peru, and Mexico. Respondents were recruited using a convenience sampling approach through advertisements on dating apps, such as Grindr (3236/16,943, 19.1%) and Hornet (6400/16,943, 37.8%), and social media platforms, such as Facebook (3374/16,943, 19.9%), Instagram (2994/16,943, 17.7%), and WhatsApp (431/16,943, 2.5%), as well as other online sources (508/16,943, 3%). Each advertisement included a link to the survey website, where participants were provided with detailed information about the study aims, procedures, and confidentiality measures. Those who provided their electronic informed consent were able to access and complete a computer-based questionnaire that included information on sociodemographics, behavior, HIV testing, and prevention. We excluded participants who reported living with HIV.

Anonymity was ensured by not collecting any personally identifiable information. The survey platform prevented multiple submissions from the same device using browser cookies and IP restrictions. Participants were permitted to select only 1 response option per question to ensure data consistency and avoid multiple selections.

### Variables

#### Outcomes

Participants were asked to report their willingness to use each of the PrEP modalities using the following question: “Considering that all PrEP modalities are available, how likely would you be to use them?” with answers recorded using a 4-point Likert scale: very unlikely, unlikely, likely, and very likely. “Very likely” answers were classified as “willing,” following the definition from previous studies [27,28]. We evaluated willingness to use the following PrEP modalities: daily oral, event-driven oral, monthly oral, bimonthly injectable in the gluteal muscle, daily topical, event-driven topical, patch, implant, infusion of monoclonal antibodies, and subcutaneous monoclonal antibodies. We also inquired whether participants were willing to use a vaccine to prevent HIV. Only factors associated with 4 PrEP modalities (daily oral, event-driven oral, monthly oral, and bimonthly injectable) were reported, as these were currently available, implemented, or under investigation in Latin America when the survey was launched.

#### Sociodemographics

For gender identity, participants answered the question “What gender do you currently identify with?” with the following possible options: cisgender man, cisgender woman, transgender woman, transgender man, queer or nonbinary, and other. Age was categorized as 18 to 24, 25 to 30, and more than 30 years. Ethnicity or race was classified as White, mixed race (*pardo* or *mestizo*), and Black, Asian, Indigenous, or other. Level of education was dichotomized as complete secondary education or less (lower education) and more than secondary education (higher education). Regarding individual monthly income, we considered the minimum wage per country in 2021 (Brazil: US $213 per month, Mexico: US $215 per month, and Peru: US $257 per month) and dichotomized as minimum wage or less (lower income) and more than minimum wage (higher income).

#### Perceived Risk of HIV Acquisition, HIV Incidence Risk Index, and HIV Testing

The perceived risk for HIV was evaluated using the question “Considering your current sexual practices, in your opinion, what is your risk of acquiring HIV in the next 12 months?” which was recategorized as low (none, low, or moderate) and high (high, or certainty of infection). The HIV Incidence Risk Index (HIRI) was constructed by combining sexual behavior and substance use variables, as described previously [29]. Scores equal to or greater than 10 were considered as “engaging in high HIV sexual exposure.” Regarding HIV testing, participants answered the question “When was the last time you took an HIV test?” which was categorized as never, 6 months or less, and more than 6 months.

### Ethical Considerations

Ethics approval for the study was obtained from the respective ethical review boards in each country. In Brazil, the study was approved by the Institutional Review Board of Instituto Nacional de Infectologia Evandro Chagas-FIOCRUZ (CAAE 82021918.0.0000.5262); in Mexico, by the Research Ethics Committee of the National Institute of Psychiatry Ramón de la Fuente Muñiz (CEI/C/038/2018); and in Peru, by the Ethical Committee for Research with Human Subjects at Universidad Peruana Cayetano Heredia (101460). All participants signed electronic informed consent form before initiating the study. No personally identifiable information was collected, except IP addresses, which were used exclusively for quality control purposes (duplicate entry prevention) and were not linked to survey responses. Data were stored on secure servers and analyzed in deidentified form. We provided no compensation to participants.

### Statistical Analysis

Statistical analyses were conducted using Stata (version 18.0; StataCorp). Frequencies and percentages of sociodemographic and behavior characteristics and HIV testing were compared by country (Brazil, Mexico, and Peru) using the *χ*^2^ test. We also described these variables according to the willingness to use 4 PrEP modalities (daily oral, event-driven oral, monthly oral, and bimonthly injectable) for each country. Descriptive analyses were based on observed data.

Missing data were assessed using Little's MCAR (missing completely at random) test, which indicated that the data were not missing completely at random (*χ*²_121_=5239; *P*<.001). Therefore, missing values in outcome and covariate variables were handled using multiple imputation by chained equations under the assumption of missing at random. Binary variables were imputed using logistic regression models and multinomial variables using multinomial logistic regression models. A total of 20 imputed datasets were generated, and parameter estimates were combined using the Rubin rules.

We conducted multivariate Poisson regression models with robust variance to estimate adjusted prevalence ratios (aPRs) and 95% CIs for willingness to use each PrEP modality (daily oral, event-driven oral, monthly oral, and bimonthly injectable). Models were adjusted for covariates selected a priori (age, country, race, education, income, HIRI score, HIV testing, and HIV risk perception). Variables were retained in the final adjusted models regardless of statistical significance.

## Results

**Figure 1:**
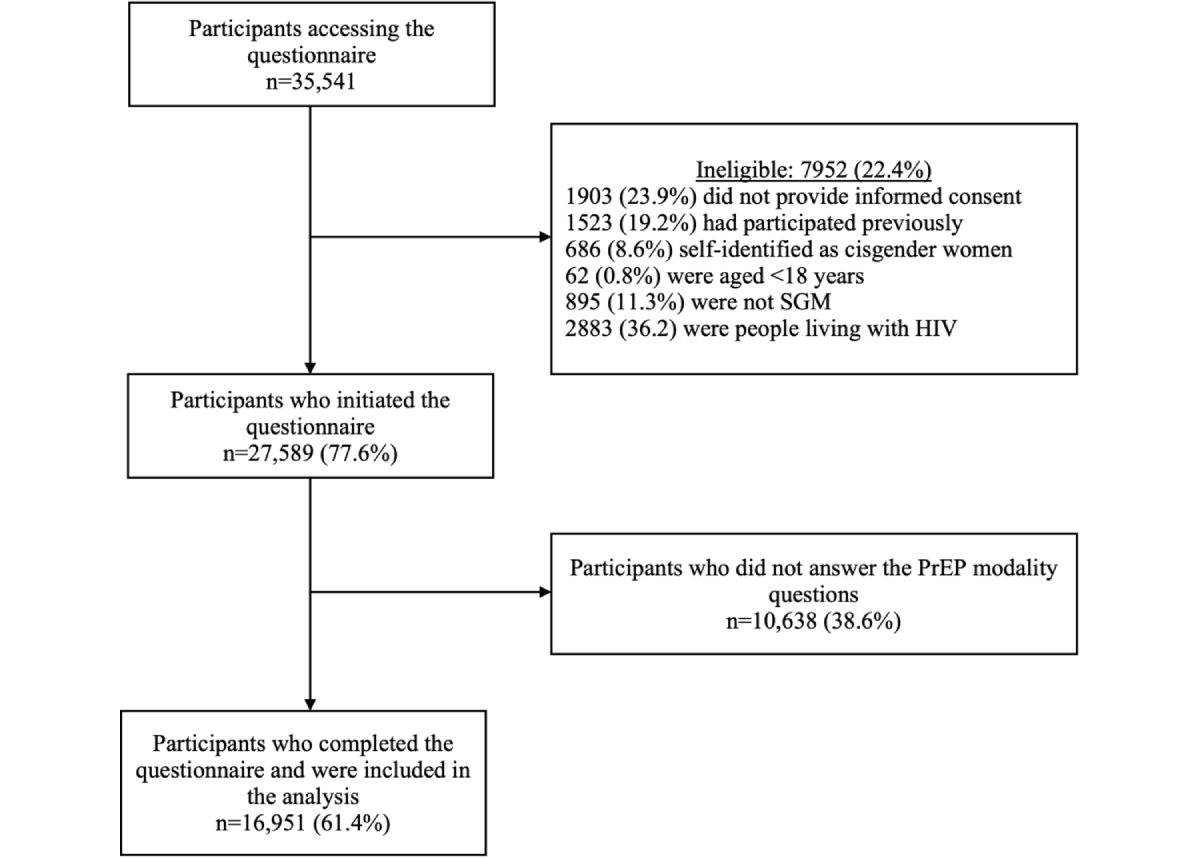
Study flowchart. PrEP: pre-exposure prophylaxis.

A total of 35,541 individuals accessed the online questionnaire, of whom 7952 (22.4%) were ineligible for the following reasons: 1903 (23.9%) did not provide informed consent, 1523 (19.2%) had participated previously, 686 (8.6%) self-identified as cisgender women, 62 (0.8%) were younger than 18 years, 895 (11.3%) were men who did not self-identify as SGM individuals, and 2883 (36.2%) reported living with HIV (Figure 1). Among 27,589 eligible individuals, 10,638 (38.6%) were excluded for not answering questions about PrEP modalities. Overall, 16,951 (61.4%) respondents were included in this analysis; 10,385 (61.3%) Brazilians, 4996 (29.5%) Mexicans, and 1570 (9.3%) Peruvians. Among these, 16,183 (95.5%) self-identified as cisgender men, 99 (0.6%) as transgender women, 60 (0.4%) as transgender men, 423 (2.5%) as nonbinary or queer persons, and 165 (1%) as other. Median age was 32 (IQR 26-39) years; almost half of the respondents (7483/16,285, 46%) self-declared mixed race, and more than three-quarters (12,627/16,509, 76.5%) received more than 1 minimum wage per month (higher income). In addition, 10,379 (61.2%) respondents were identified as having high HIV sexual exposure according to the HIRI, 1961 (11.6%) had never been tested for HIV, and 6688 (39.6%) had their last HIV test more than 6 months ago.

Study population characteristics were compared by country (Table 1). More Peruvians (164/1570, 10.5%) self-identified as transgender or nonbinary persons compared with Brazilians (224/10,384, 2.2%) and Mexicans (379/4996, 7.6%) and were aged 18 to 24 years (Peru: 716/1570, 45.6%; Mexico: 1057/4996, 21.2%; Brazil: 1304/10,385, 12.6%). Regarding race, most Brazilians (5873/10,385, 56.5%) self-defined as White, while most Mexicans (3249/4330, 75%) and Peruvians (1109/1570, 70.6%) self-identified as mixed race. Most individuals in Brazil and Mexico received more than minimum wage monthly (8179/10,385, 78.8% and 3738/4729, 79%, respectively) compared with Peru (710/1395, 50.9%). In addition, more Peruvians (180/1194, 15.1%) perceived themselves to be at high risk of acquiring HIV compared with Brazilians (964/10,385, 9.3%) and Mexicans (439/4996, 8.8%); conversely, more Brazilians (6676/10,385, 64.3%) had high HIV sexual exposure according to HIRI scale compared with Mexicans (2965/4996, 59.4%) and Peruvians (738/1570, 47%). Additionally, a higher proportion of Peruvians (328/1550, 21.2%) had never been tested for HIV compared with Mexicans (757/4972, 15.2%) and Brazilians (876/10,385, 8.4%).

**Figure 2:**
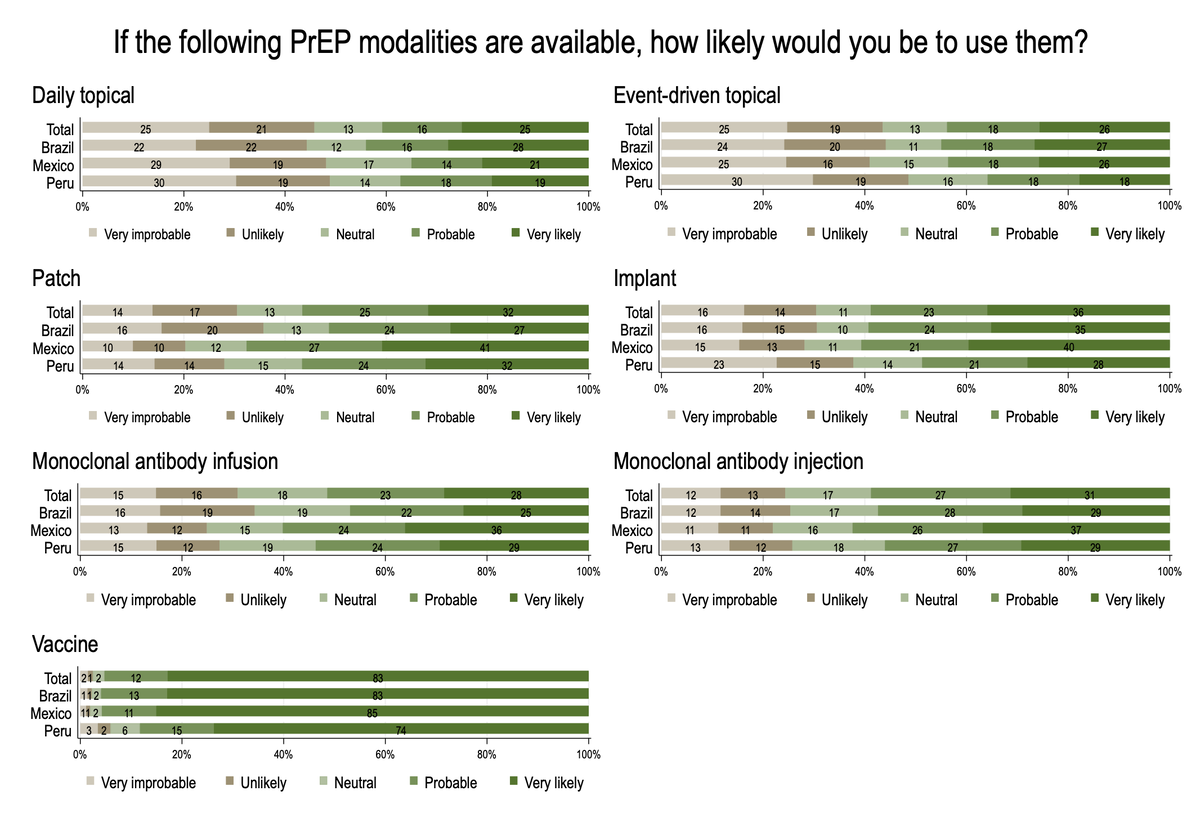
Willingness to use pre-exposure prophylaxis (PrEP) modalities and an HIV vaccine among sexual and gender minority individuals in Brazil, Mexico, and Peru.

Regarding PrEP modalities, most of the respondents were willing to use monthly oral PrEP (12,621/16,951, 74.4%), daily oral PrEP (11,153/16,951, 65.8%), and bimonthly injectable PrEP (10,212/16,951, 60.2%), while only 38% (6442/16,951) were willing to use event-driven PrEP. Other PrEP modalities received lower willingness levels: 25.1% (4109/16,398) for daily topical, 25.6% (4202/16,398) for event-driven topical, 31.7% (5204/16,399) for a patch, 35.9% (5885/16,398) for an implant, 28.4% (4660/16,397) for infusion monoclonal antibodies, and 31.3% (5139/16,398) for subcutaneous monoclonal antibodies. Additionally, 82.8% (13,579/16,398) of participants reported willingness to use an HIV vaccine. Figure 2 shows the percentages of willingness to use these less common PrEP modalities and an HIV vaccine in each country.

We also found country-level differences in the willingness to use at least one of the 4 PrEP modalities evaluated (daily oral, event-driven oral, monthly oral, and bimonthly injectable). Mexicans reported the highest overall willingness (4449/4903, 90.7%), followed by Brazilians (8965/10,034, 89.4%) and Peruvians (1195/1423, 84%). Within each country, there were substantial differences in the willingness to use each of these 4 PrEP modalities. Peruvians were less willing to use daily oral (799/1506, 53.1%), monthly oral (830/1328, 62.5%), and bimonthly injectable (610/1328, 45.9%) compared with Brazilians (daily oral: 6082/9106, 66.8%; monthly oral: 7640/10,197, 74.9%; bimonthly injectable: 6450/10,197, 63.3%) and Mexicans (daily oral: 3242/4784, 67.8%; monthly oral: 3728/4875, 76.5%; bimonthly injectable: 2805/4875, 57.5%). Willingness to use event-driven PrEP was lower for Brazilians (3162/8761, 36.1%).

In all 3 countries, cisgender MSM were more willing to use monthly oral PrEP compared with transgender or nonbinary participants (Table 2). In Brazil (4376/5780, 75.7%) and Mexico (619/788, 78.6%), White individuals were more willing to use monthly oral PrEP, while in Peru (610/951, 64.1%), mixed-race individuals showed higher willingness. In Mexico (469/788, 59.5%) and Peru (80/168, 47.6%), White participants were more willing to use bimonthly injectable PrEP, whereas in Brazil (884/6450, 64.7%), Black, Asian, or Indigenous individuals showed greater willingness. In Brazil and Mexico, daily oral PrEP was more preferred among individuals with lower education (2165/6082, 68.7% and 983/1429, 68.8%, respectively) and lower income (1368/2032, 67.3% and 664/969, 68.5%), while in Peru, it was more preferred among participants with higher education (519/958, 54.2%) and income (378/671, 56.3%). In Brazil and Mexico, participants who had an HIV test within the past 6 months showed greater willingness to use monthly oral PrEP (4148/5462, 75.9% and 1576/2010, 78.4%, respectively), while in Peru, this modality was more preferred by people who had an HIV test more than 6 months ago (313/491, 63.8%).

In the multivariable analysis (Table 3), willingness to use each of the 4 evaluated PrEP modalities was positively associated with high HIV self-perceived risk and higher HIRI scores. Peruvian respondents showed lower willingness to use 3 modalities (daily oral: aPR=0.81, 95% CI 0.75-0.87; monthly oral: aPR=0.84, 95% CI 0.78-0.91; and injectable: aPR=0.76, 95% CI 0.69-0.83). Mexican and Peruvian respondents reported higher willingness to use event-driven oral PrEP (aPR=1.14, 95% CI 1.08-1.22 and aPR=1.11, 95% CI 1.01-1.22, respectively) compared with Brazilian respondents. Having secondary education or less was associated with lower willingness to use monthly oral and bimonthly injectable PrEP (aPR=0.93, 95% CI 0.89-0.97 and aPR=0.94, 95% CI 0.90-0.99, respectively). Having a minimum wage or less was associated with lower willingness for bimonthly injectable PrEP (aPR=0.95, 95% CI 0.90-1.00). Never having been tested for HIV and testing for more than 6 months ago were associated with lower willingness to use daily oral PrEP (aPR=0.89, 95% CI 0.83-0.95 and aPR=0.95, 95% CI 0.91-0.99, respectively) and bimonthly injectable PrEP (aPR=0.80, 95% CI 0.74-0.86 and aPR=0.90, 95% CI 0.86-0.94, respectively).

**Table 1:** Characteristics of an online sample of sexual and gender minority individuals (N=16,951) in Brazil (n=10,385, 61.3%), Mexico (n=4996, 29.5%), and Peru (n=1570, 9.3%) in 2021.

Characteristics			Total (n=16,951), n (%)		Brazil (n=10,385), n (%)		Mexico (n=4996), n (%)		Peru (n=1570), n (%)		*P* value	
**Gender identity (n=16,950)**												<.001
	Cisgender men	16,183 (95.5)		10,160 (97.8)		4617 (92.4)		1406 (89.5)				
	Transgender or nonbinary persons	767 (4.5)		224 (2.2)		379 (7.6)		164 (10.5)				
**Age (years; n=16,951)**												<.001
	18-24	3077 (18.1)		1304 (12.6)		1057 (21.2)		716 (45.6)				
	25-30	4604 (27.2)		2712 (26.1)		1456 (29.1)		436 (27.8)				
	>30	9270 (54.7)		6369 (61.3)		2483 (49.7)		418 (26.6)				
**Race (n=16,285)**												<.001
	White	6879 (42.2)		5873 (56.5)		808 (18.7)		198 (12.6)				
	Mixed race (*pardo* or *mestizo*)	7483 (46)		3125 (30.1)		3249 (75)		1109 (70.6)				
	Black, Asian, or Indigenous	1923 (11.8)		1387 (13.4)		273 (6.3)		263 (16.8)				
**Education level (n=16,951)**												<.001
	Less than or equal to secondary education	5455 (32.2)		3419 (32.9)		1474 (29.5)		562 (35.8)				
	More than secondary education	11,496 (67.8)		6966 (67.1)		3522 (70.5)		1008 (64.2)				
**Monthly income (n=16,509)**												<.001
	Less than or equal to 1 minimum wage	3882 (23.5)		2206 (21.2)		991 (21)		685 (49.1)				
	More than 1 minimum wage	12,627 (76.5)		8179 (78.8)		3738 (79)		710 (50.9)				
**HIV risk perception (n=16,575)**												<.001
	None, low, or moderate	14,992 (90.5)		9421 (90.7)		4557 (91.2)		1014 (84.9)				
	High	1583 (9.5)		964 (9.3)		439 (8.8)		180 (15.1)				
**HIRI^a^** **(n=16,951)**												<.001
	Low risk	6572 (38.8)		3709 (35.7)		2031 (40.6)		832 (53)				
	High risk	10,379 (61.2)		6676 (64.3)		2965 (59.4)		738 (47)				
**Last HIV test (n=16,907)**												<.001
	Never	1961 (11.6)		876 (8.4)		757 (15.2)		328 (21.1)				
	Less than or equal to 6 months	8258 (48.8)		5543 (53.4)		2055 (41.3)		660 (42.6)				
	More than 6 months	6688 (39.6)		3966 (38.2)		2160 (43.4)		562 (36.3)				

^a^HIRI: HIV Incidence Risk Index.

**Table 2:** Characteristics of sexual and gender minority individuals willing to use each pre-exposure prophylaxis (PrEP) modality by country, 2021 (N=16,951).

Characteristics				Brazil (PrEP modality)									Mexico (PrEP modality)									Peru (PrEP modality)									
				Daily, n (%)		ED^a^, n (%)		Monthly, n (%)		Inject n, (%)		Daily, n (%)			ED, n (%)		Monthly, n (%)		Inject, (%)		Daily, n (%)			ED, n (%)		Monthly, n (%)		Inject, n (%)			
**Willingness to use**																															
		Yes	6082 (66.8)		3162 (36.1)		7640 (74.9)		6450 (63.3)		3242 (67.8)			1958 (41)		3728 (76.5)		2805 (57.5)		799 (53.1)			584 (39.9)		830 (62.5)		610 (45.9)				
**Gender identity**																															
	Cisgender men		5953 (66.9)		3088 (36.1)		7477 (75)		6316 (63.3)		2995 (67.8)			1812 (41.1)		3473 (77.2)		2603 (57.8)		723 (53.6)			531 (40.1)		761 (63.3)		557 (46.3)				
	Transgender or nonbinary		129 (62.6)		74 (37.2)		163 (73.8)		134 (60.6)		247 (66.9)			146 (40)		255 (68)		202 (53.9)		76 (48.1)			53 (37.6)		69 (55.2)		53 (42.4)				
**Age (years)**																															
	18-24		817 (66.8)		401 (34.1)		947 (74)		763 (59.6)		724 (70.2)			431 (43.3)		783 (76.2)		533 (51.9)		359 (51.4)			270 (40.1)		377 (62.2)		252 (41.6)				
	25-30		1689 (70.1)		876 (37.8)		2017 (75.9)		1706 (64.2)		1008 (70.9)			599 (42.5)		1098 (77.1)		855 (60)		217 (52.7)			161 (39.7)		240 (63)		194 (50.9)				
	>30		3576 (65.3)		1885 (35.8)		4676 (74.7)		3981 (63.6)		1510 (64.7)			928 (39.1)		1847 (76.2)		1417 (58.5)		223 (56.5)			153 (39.8)		213 (62.5)		164 (48.1)				
**Race**																															
	White		3296 (64.6)		1755 (35.6)		4376 (75.7)		3609 (62.4)		510 (67.1)			327 (42.3)		619 (78.6)		469 (59.5)		92 (48.2)			78 (42.4)		98 (58.3)		80 (47.6)				
	Mixed race (*pardo or mestizo*)		1915 (69.1)		946 (35.8)		2275 (74.6)		1957 (64.2)		2107 (67.6)			1280 (41.1)		2456 (77.5)		1872 (59.1)		574 (54)			423 (40.4)		610 (64.1)		435 (45.7)				
	Black, Asian, or Indigenous		871 (70.9)		461 (38.7)		989 (72.4)		884 (64.7)		186 (71.5)			126 (47.6)		193 (71.8)		138 (51.3)		133 (53)			83 (35.6)		122 (58.4)		95 (45.5)				
**Education level**																															
	Secondary education or less		2165 (68.7)		1057 (35.3)		2368 (71)		1976 (59.3)		983 (68.8)			587 (42)		1054 (73.2)		784 (54.4)		280 (51.1)			223 (43.1)		286 (59.3)		204 (42.3)				
	More than secondary education		3917 (65.8)		2105 (36.5)		5272 (76.8)		4474 (65.2)		2259 (67.3)			1371 (40.6)		2674 (77.9)		2021 (58.8)		519 (54.2)			361 (38.2)		544 (64.3)		406 (48)				
**Monthly income**																															
	Minimum wage or less		1368 (67.3)		708 (36.7)		1543 (71.7)		1256 (58.3)		664 (68.5)			401 (42.6)		721 (74.6)		520 (53.8)		340 (51.1)			255 (39.9)		336 (58.7)		249 (43.5)				
	More than minimum wage		4714 (66.6)		2454 (35.9)		6097 (75.8)		5194 (64.6)		2428 (68.2)			1462 (40.8)		2810 (77)		2153 (59)		378 (56.3)			274 (41.1)		409 (66.8)		312 (51)				
**HIV risk perception**																															
	None, low, or moderate		5345 (64.9)		2809 (35.4)		6851 (74.1)		5730 (62)		2887 (66)			1734 (39.8)		3364 (75.7)		2501 (56.3)		517 (52.8)			409 (40.3)		633 (62.4)		454 (44.8)				
	High		737 (84.9)		353 (42.3)		789 (83.2)		720 (76)		355 (87)			224 (53)		364 (84.9)		304 (70.9)		131 (79.4)			90 (50)		125 (69.4)		99 (55)				
**HIRI^b^**																															
	Low risk		1972 (56.8)		1114 (33.7)		2523 (69.7)		1950 (53.9)		1190 (59.8)			746 (38.6)		1423 (72)		1018 (51.5)		367 (45.8)			277 (38.2)		355 (60.2)		249 (42.2)				
	High risk		4110 (72.9)		2048 (37.5)		5117 (77.8)		4500 (68.4)		2052 (73.4)			1212 (42.6)		2305 (79.5)		1787 (61.7)		432 (61.4)			307 (41.6)		475 (64.4)		361 (48.9)				
**Last HIV test**																															
	Never		545 (62.7)		280 (34.7)		597 (70.8)		443 (52.6)		470 (62.7)			272 (38.6)		537 (74.1)		350 (48.3)		130 (40.6)			108 (37.5)		143 (58.6)		80 (32.8)				
	6 months or less		2983 (69.2)		1544 (37.1)		4148 (75.9)		3684 (67.5)		1363 (73.1)			851 (43)		1576 (78.4)		1299 (64.6)		362 (59.2)			262 (41.7)		371 (63.6)		295 (50.6)				
	More than 6 months		2554 (65)		1338 (35.2)		2895 (74.4)		2323 (59.7)		1398 (65.1)			830 (40)		1600 (75.5)		1144 (54)		298 (53.8)			207 (38.9)		313 (63.8)		232 (47.3)				

^a^ED: event-driven.

^b^HIRI: HIV Incidence Risk Index.

**Table 3:** Factors associated with willingness to use each pre-exposure prophylaxis modality among sexual and gender minorities in Brazil, Mexico, and Peru in 2021.

Characteristics			Daily oral, aPR^a^ (95% CI)		Event-driven, aPR (95% CI)		Monthly oral, aPR (95% CI)		Injectable, aPR (95% CI)
**Country**									
	Brazil	Reference		Reference		Reference		Reference	
	Mexico	1.02 (0.97-1.06)		*1.14 (1.08-1.22)* ^b^		1.02 (0.98-1.07)		*0.93 (0.89-0.98)*	
	Peru	*0.81 (0.75-0.87)*		*1.11 (1.01-1.22)*		*0.84 (0.78-0.91)*		*0.76 (0.69-0.83)*	
**Age (years)**									
	18-24	1.01 (0.96-1.07)		1.02 (0.94-1.10)		1.04 (0.98-1.11)		0.95 (0.89-1.01)	
	25-30	1.03 (0.99-1.08)		1.04 (0.98-1.11)		1.01 (0.97-1.06)		1.00 (0.95-1.05)	
	>30	Reference		Reference		Reference		Reference	
**Race**									
	White	Reference		Reference		Reference		Reference	
	Mixed (Black and *pardo*)	1.04 (0.99-1.09)		0.99 (0.93-1.05)		0.99 (0.95-1.04)		1.02 (0.98-1.07)	
	Black, Asian, or Indigenous	1.07 (1.00-1.14)		1.05 (0.96-1.14)		0.95 (0.89-1.01)		1.01 (0.95-1.08)	
**Education level**									
	Secondary education or less	1.02 (0.98-1.07)		0.98 (0.92-1.04)		*0.93 (0.89-0.97)*		*0.94 (0.90-0.99)*	
	More than secondary education	Reference		Reference		Reference		Reference	
**Monthly income**									
	Minimum wage or less	0.98 (0.94-1.03)		1.02 (0.95-1.09)		0.96 (0.92-1.01)		*0.95 (0.90-1.00)*	
	More than minimum wage	Reference		Reference		Reference		Reference	
**HIV risk perception**									
	None, low, or moderate	Reference		Reference		Reference		Reference	
	High	*1.25 (1.18-1.32)*		*1.21 (1.12-1.32)*		*1.10 (1.04-1.17)*		*1.17 (1.10-1.25)*	
**HIRI^c^**									
	Low risk	Reference		Reference		Reference		Reference	
	High risk	*1.22 (1.17-1.27)*		*1.08 (1.02-1.14)*		*1.10 (1.06-1.14)*		*1.21 (1.16-1.26)*	
**Last HIV test**									
	Never	*0.89 (0.83-0.95)*		0.92 (0.85-1.00)		0.97 (0.91-1.03)		*0.80 (0.74-0.86)*	
	6 months or less	Reference		Reference		Reference		Reference	
	More than 6 months	*0.95 (0.91-0.99)*		0.95 (0.90-1.01)		0.99 (0.96-1.03)		*0.90 (0.86-0.94)*	

^a^aPR: adjusted prevalence ratio.

^b^Italicization indicates statistical significance (*P*<.05).

^c^HIRI: HIV Incidence Risk Index.

## Discussion

### Principal Findings

In this paper, we provide information about willingness to use PrEP modalities and an HIV vaccine among SGM individuals from 3 countries in Latin America. Overall, participants were more interested in monthly oral PrEP, with daily oral and bimonthly injectable PrEP also being widely accepted and event-driven oral PrEP being the least preferred. Most participants reported willingness to use an HIV vaccine. Participants perceiving themselves at higher HIV risk and engaging in higher HIV sexual exposure were more willing to use all 4 PrEP modalities. Individuals with lower education were less willing to use long-acting PrEP (monthly oral and bimonthly injectable). Participants who had never tested for HIV or had been tested more than 6 months ago were less willing to use daily oral PrEP and bimonthly injectable. Our study provides insight into PrEP preferences among SGM individuals in Latin America and can guide the implementation of new PrEP modalities in prevention programs.

Higher willingness to use monthly oral and bimonthly injectable PrEP indicates a preference for long-acting formulations among SGM individuals in Latin America. In a discrete-choice experiment conducted in Brazil, SGM individuals preferred long-acting PrEP requiring less frequent dosing (monthly, bimonthly, or annually) as long as efficacy and side effects were similar or lower than those of oral PrEP [30]. Additionally, participants did not show a stronger preference for annual dosing compared with monthly or bimonthly dosing [30]. Previous studies identified injectable PrEP as the most preferred modality; however, they did not consider monthly oral PrEP as an option [31-33]. Currently, monthly oral PrEP is not available, although MK-8527, a novel investigational nucleoside reverse transcriptase translocation inhibitor, is under evaluation as an alternative to daily oral PrEP [34]. Two phase 3 clinical trials (EXPrESSIVE 10 and 11) are investigating the efficacy, safety, and tolerability of MK-8527 monthly compared with daily oral PrEP (FTC/TDF) among cisgender women and SGM individuals in several regions, including Latin America [35].

Long-acting injectables have been considered highly acceptable among SGM populations for HIV prevention [36]. This is corroborated by findings from a survey conducted among 3665 Brazilian SGM individuals in 2024 [37]. In the ImPrEP CAB Brasil study, most participants (83%) chose CAB-LA over daily oral PrEP [38]. Long-acting injectables offer potential advantages, such as reducing the frequency of clinic visits and improving adherence compared with daily oral PrEP [39]. Our study also revealed that individuals with lower education were less willing to use long-acting PrEP formulations, similar to findings related to injectable PrEP in Brazil [31]. This suggests that people using PrEP need accessible information to better understand the important features of these modalities.

Daily oral PrEP was also widely accepted; however, Peruvians were less likely to be willing to use this modality compared with Brazilians and Mexicans. This result is consistent with results from the ImPrEP study, which reported lower persistence and adherence to daily oral PrEP among MSM and transgender women from Peru compared with Brazil [11,12]. Possible contributing factors included low awareness of oral PrEP, HIV-related stigma during sexually transmitted infection clinic visits, and concerns about medication efficacy and side effects [27]. These concerns may be more noticeable with newer PrEP modalities, particularly long-acting formulations, due to the lack of information and perceived loss of autonomy in administration, which may contribute to a preference for the daily oral modality [33,40]. Additionally, familiarity with and routine use of a daily oral pill could also have contributed to this preference [40].

Differences among countries may also reflect structural and contextual barriers. Brazil and Mexico incorporated daily oral PrEP into their public health systems in 2017 and 2021, respectively, and have since implemented large-scale, community-supported programs that promote familiarity with and confidence in PrEP [8,41]. In contrast, the Peruvian Ministry of Health initiated offering oral PrEP to populations at high vulnerability for HIV in 2023 [9]. This delayed implementation, with limited-service availability, may have reduced visibility and accessibility among potential users. Strengthening PrEP implementation in Peru through decentralized service delivery, stigma reduction initiatives, and targeted communication campaigns could help improve uptake and promote more equitable access across the region.

Event-driven oral PrEP was the least acceptable among our participants. This finding aligns with previous studies [31,32], except for a study conducted in the United States, in which MSM reported that most of condomless anal sex events are either infrequent or can be anticipated [42]. In contrast, participants in the ImPrEP study showed low interest in switching from daily oral to event-driven oral PrEP [43]. Reasons included that fewer than 25% reported having sex less than 2 days per week (indicating infrequent sex), most were satisfied with the daily regimen, considered event-driven PrEP a difficult regimen to follow, and had concerns about its efficacy and anxiety about their own HIV risk [43].

Individuals who perceived themselves at high risk for HIV and who were engaging in high HIV sexual exposure were more likely to use any PrEP modality. A high perceived risk of HIV has been identified as a facilitator of PrEP acceptability and willingness to use it [44-46]. For example, in Mexico, transgender women reported a high willingness to use daily oral PrEP (95.5%) if they had high HIV risk perception [47]. Additionally, Torres et al [31] reported that higher HIRI scores increased the willingness of use injectable PrEP, while lower HIRI scores were associated with a preference for event-driven PrEP in Brazil and Mexico. In another Latin American study, Assaf et al [48] found that a higher risk for HIV was associated with PrEP awareness in Brazil but not among MSM in Mexico and Peru. Importantly, a study from Brazil [27] found that PrEP awareness was associated with willingness to use it, indicating the importance of continuous education campaigns about HIV prevention, including current and future PrEP modalities.

PrEP modalities involving topical agents, patches, implants, and monoclonal antibodies received lower willingness to use compared with the oral and injectable modalities. In contrast, HIV vaccines were widely accepted by participants. Many of these alternative PrEP formulations are still in the clinical research phase [20,49], while no HIV vaccine has been efficacious to date. Limited awareness of the options still under study could have generated distrust. However, previous study from South Africa have reported high acceptability of topical agents among different populations, including SGM individuals [49]. Although there is currently no approved HIV vaccine, the MOSAICO study (HPX3002/HVTN706) enrolled Latin American participants before being discontinued due to lack of efficacy [50]. Nevertheless, previous studies from Brazil found a high willingness to use a hypothetical effective HIV vaccine, even if it was not free of charge [51,52].

We acknowledge that this study has certain limitations. Participants were recruited through dating apps and social media platforms and accessed the online survey using smartphones or other internet-connected devices. This recruitment strategy was appropriate for reaching SGM communities but may have introduced selection bias by excluding individuals with limited digital access, meaning that our sample may not be representative of all SGM individuals in Latin America, Brazil, Mexico, or Peru. Additionally, the cross-sectional design of our study limited our ability to establish causality. Notably, when the data were collected in 2021, only daily oral PrEP was available in these countries. However, oral PrEP information was more widely disseminated in Brazil, where it had been included in their public health care system since 2017 [41]. Finally, all responses were based on participants’ self-reports, and social desirability bias may have occurred; however, online anonymous data collection may have reduced this bias. It is also important to note that “willingness” reflects hypothetical acceptability and may not directly translate into actual intention or behavior, which should be considered when interpreting these findings.

### Conclusions

We found that SGM individuals from Latin America were more willing to use long-acting PrEP, including monthly oral and bimonthly injectable PrEP, but daily oral PrEP was also highly accepted. Further research and education are needed to better understand and address the gaps in knowledge about prevention modalities. The availability of additional choices to better address the prevention needs of SGM populations could empower individuals to use these methods. As additional PrEP modalities are included in HIV prevention programs, the development of accessible tools and community-based strategies will be essential to support informed PrEP choices and ensure equitable implementation across the region.

## Abbreviations

aPR: adjusted prevalence ratio
CAB-LA: long-acting injectable cabotegravir
FTC/TDF: emtricitabine 200 mg and tenofovir disoproxil fumarate 300 mg
HIRI: HIV Incidence Risk Index
LEN: long-acting injectable lenacapavir
MSM: men who have sex with men
PrEP: pre-exposure prophylaxis
SGM: sexual and gender minority
WHO: World Health Organization

## Data Availability

The datasets generated or analyzed during this study are available from the corresponding author on reasonable request.
